# Necrotizing Fasciitis and Mediastinitis after Wisdom Tooth Extraction: A Case Report

**DOI:** 10.21980/J8XW7K

**Published:** 2020-10-15

**Authors:** Jennifer Edwards, Ryan Fisher, Amrita Vempati

**Affiliations:** * Creighton University School of Medicine Phoenix Program, Maricopa Medical Center, Department of Emergency Medicine, Phoenix, AZ

## Abstract

Necrotizing soft tissue infections with mediastinal extension are exceedingly rare. This submission provides dynamic and static visual stimuli (CT images) and a case report describing the history of present illness, initial ED presentation and inpatient course of a 33-year-old man who developed facial necrotizing fasciitis complicated by mediastinitis secondary to odontogenic infection following wisdom tooth extraction. Discussion of the key features of this rare presentation, the diagnostic and therapeutic approaches to making the diagnosis (including CT), and the role of the Emergency Physician, are highlighted.

**Topics:**

Necrotizing fasciitis, necrotizing mediastinitis, odontogenic infection, CT scan.

**Figure:**
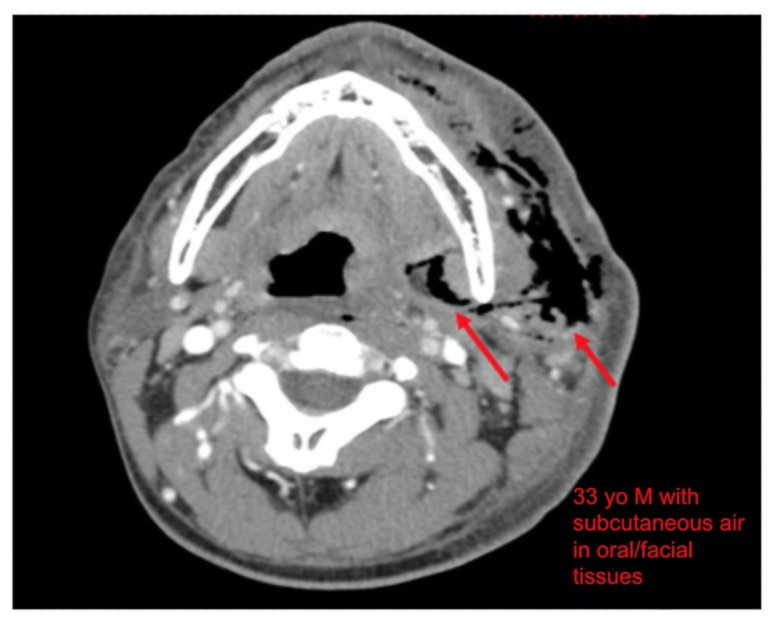

**Figure:**
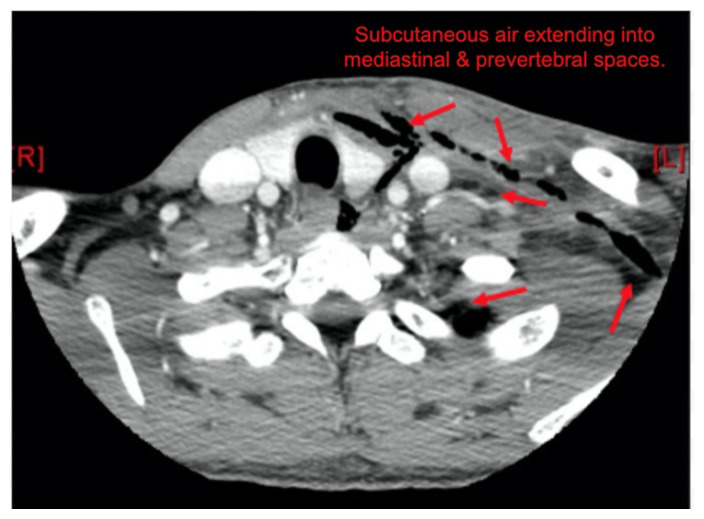
Axial CT Video Link: https://youtu.be/aqTOSnGU0vg

## Introduction

Necrotizing soft tissues infections (NSTI) can present with symptoms similar to cellulitis such as swelling, erythema and pain; however, as the infection progresses, the symptoms may consist of crepitus, bullae or blisters.^1^ With the progression of infection, the morbidity and mortality sharply increases. Patients who were taken to surgery within 6 hours of presentation had 19% mortality compared to 32% in patients who were taken to surgery after 6 hours.^2^ NSTIs are classified into 4 types based on the organism causing the infection. Type 1 is caused by mixed anaerobes and aerobes, Type 2 often by Group A *Streptococcus* (GAS), Type 3 often by *Vibrio* species, and Type 4 by fungi. Type I NSTI infection is the most common followed by Type 2.^3^ However, the Centers for Disease Control only monitors NSTI which is caused by GAS and reported 7.6 per 100,000 in 2018, which is underreported.^4^ The actual number of cases may be higher if all types were reported. However, it still remains a rare infection with devastating complications that needs to be recognized quickly. This report illustrates a rare case of cervical necrotizing fasciitis complicated by necrotizing mediastinitis. Static images and video of the CT scan demonstrate the extent of this patient’s subcutaneous air. While this case, and most others of necrotizing soft tissues infections, present late in the clinical course, this report will discuss the utility of laboratory, radiographic, and clinical scoring tools.

## Presenting concerns and clinical findings

A 33-year-old male with no significant past medical history presented to the emergency department with left-sided facial swelling. Two weeks prior, the patient had his wisdom teeth extracted in Mexico. He noticed slight facial swelling a week after the procedure; however, the night prior he had severe pain and used cocaine and methamphetamine to help alleviate the pain. When he woke up in the morning, swelling had significantly worsened. In addition, he noticed spreading redness with increasing pain. The patient denied fevers, and triage vitals were significant only for tachycardia of 144.

On physical exam, the patient was noted to have extensive swelling over the left side of his face and neck, extending from the superior lid of the left eye to the left distal neck. There was induration on the superior eye lid and crepitus over the left angle of the mandible. Examination of extraocular movements were difficult due to the extent of facial edema; however, patient reported no pain with eye movements. During examination, the patient tolerated his secretions well with no noted voice changes, but trismus was present. In addition, he had multiple involuntary facial muscle spasms despite being fully awake and alert. He had elevation of the jaw, raising suspicion for Ludwig’s angina. Due to high suspicion for infection from the clinical exam and history, the patient was empirically started on clindamycin for odontogenic source coverage along with fluids for tachycardia and sepsis.

## Significant findings

Labs were notable for leukocytosis to 18.5 with a bandemia of 26, lactic acidosis of 2.9 with resulting compensated anion gap metabolic acidosis (anion gap of 20, bicarbonate of 20), and an acute kidney injury with a creatine of 3.72.

Computer tomography (CT) imaging of soft tissues of the neck and of the chest/abdomen/pelvis revealed extensive swelling and subcutaneous air (see red arrows) on the left side of the face and neck extending to the left shoulder, as well as parapharyngeal/retropharyngeal spaces and posterior/superior mediastinum.

## Patient Course

Throughout his ED course, the patient’s mental status declined; however, he remained hemodynamically stable and independently protected his airway. From the crepitus noted on physical exam, there was suspicion for a necrotizing infection, and this was a high concern with the patient’s clinical decompensation. A quick review of the CT images by the ED treatment team reveal diffuse subcutaneous air in the patient’s face. With findings consistent with a NSTI, the antibiotics were expanded to piperacillin/tazobactam and linezolid. Due to the acute clinical change, Burn Surgery, who manages NSTIs at our hospital, was consulted for emergent surgery. During this time, the CT was formally read by the Radiologist revealing the extent of the infection to necrotizing mediastinitis.

Given the location of the infection and the high-risk airway, the patient was intubated in the operating room with anesthesia and otolaryngology present. Surgical exploration revealed the patient to have left neck necrotizing fasciitis from odontogenic infection, extending through masseter space and tracking along carotid sheath and prevertebral space. The submandibular and parotid glands were also necrotic. Wound cultures eventually grew *Streptococcus constellatus* and parenteral antibiotics were narrowed to cefazolin.

After initial surgical re-exploration, a decision was made to place local antibiotic beads, coated with vancomycin and gentamicin, due to the appearance of the facial muscle tissue. However, these beads were dislodged post-operatively on day one. The patient continued to be managed with local wound care and parenteral antibiotics.

Over the twenty-day hospital course, he required a total of thirteen procedures, and was eventually discharged with oral antibiotics. The patient followed up with Plastic Surgery and Burn Surgery once discharged. He was able to regain full function of his extremities, had no limitations on daily activities, and suffered no limitations with swallowing or speaking. However, the patient continued to struggle with a drug addiction to methamphetamines and was lost to follow up five months after hospital discharge.

## Discussion

Necrotizing soft tissue infections (NSTI) are a complex pathology which carries a high morbidity and mortality owing to the difficulty of its clinical diagnosis.^5^ While there are many risk factors including diabetes, intravenous drug abuse (IVDA), immunocompromise, alcohol abuse, and nonsteroidal anti-inflammatory drugs, otherwise healthy patients can also develop NSTI.^5^–^8^ The hard signs of NSTI include presence of bullae, skin ecchymosis, presence of gas in the tissues or in radiographic evaluation and cutaneous anesthesia. These signs, however, are only present in 7–44% of the cases.^9^ Often, a NSTI can be difficult to distinguish from a severe cellulitis, and cervical NSTIs even harder to diagnose. A review of literature for necrotizing cervical infections reveals that initial presentations vary wildly, but are “hallmarked by... pain disproportionate to or in the absence of clinical findings.”^10^,^11^ Of the 1235 reviewed patients with cervical NSTI, 47% were from an odontogenic source.^10^ The disease progression is very rapid with NSTIs, as described in this patient. For any patient describing rapidly progressing symptoms of infection, NSTI must be very high in the differential diagnosis. Radiographic evaluation may not be very helpful as well. Although subcutaneous air, when seen on radiographic imaging, such as this case, is pathognomonic, it is only evident on 60% of plain radiographs and 56.8% of CT.^10^

In an effort to assist with diagnosis, the laboratory risk indicator for necrotizing fasciitis (LRINEC) score was developed as a decision tool in 2004.^15^ The score, based on six criteria (C-reactive protein, white blood cell count, hemoglobin, sodium, creatinine, and glucose), classified patients as low-, moderate-, or high-risk for a necrotizing infection. However, there are multiple concerns about the utility of the LRINEC score. Specifically, in the original study population, 10% of patients who received a low-risk score were found to have an intraoperative diagnosis of necrotizing fasciitis. Additionally, the external validation study could not reproduce the same sensitivity, specificity, negative predictive value (NPV), or positive predictive value (PPV).^16^ Further validation studies and studies with expanded testing criteria have shown sensitivity, specificity, NPV and PPV closer to the original 2004 study.^17^ One study applying the LRINEC score specifically for cervical necrotizing infections demonstrated that a LRINEC score ≥ 6 had poor statistical validity.^18^

With 40% of cervical NSTI lacking pathognomonic radiographic findings and a poorly validated clinical decision tool, the diagnosis of the infection still lies with clinical suspicion. This can be difficult if the clinical presentation or physical exam is not extreme, which will allow time for the disease process to develop complications, such as mediastinitis. Because it can be difficult to make this diagnosis early, any clinical suspicion should prompt early antibiotic administration and surgical consultation.

The gold standard of management is early surgical debridement, with aggressive surgical management as the “single most important treatment modality for determining patient outcome.”^12^ Delaying surgery even 24 hours is an independent mortality risk factor.^13^,^14^ Empiric antibiotic choice may be dictated by a hospital’s antibiogram. The Infectious Diseases Society of America recommends empiric therapy of vancomycin or linezolid plus piperacillin-tazobactam or a carbapenem; or plus ceftriaxone and metronidazole.^19^ Despite medical interventions and differential surgical approaches, the mortality of cervical necrotizing fasciitis complicated by descending necrotizing mediastinitis remains 40%.^20^

## Supplementary Information










