# Supplementary figures and images for: Necrotizing Fasciitis and Mediastinitis after Wisdom Tooth Extraction: A Case Report
Source: J Educ Teach Emerg Med. 2020 Oct 15;5(4):V1–5. doi: 10.21980/J8XW7K (PMC10332516; doi:10.21980/J8XW7K)

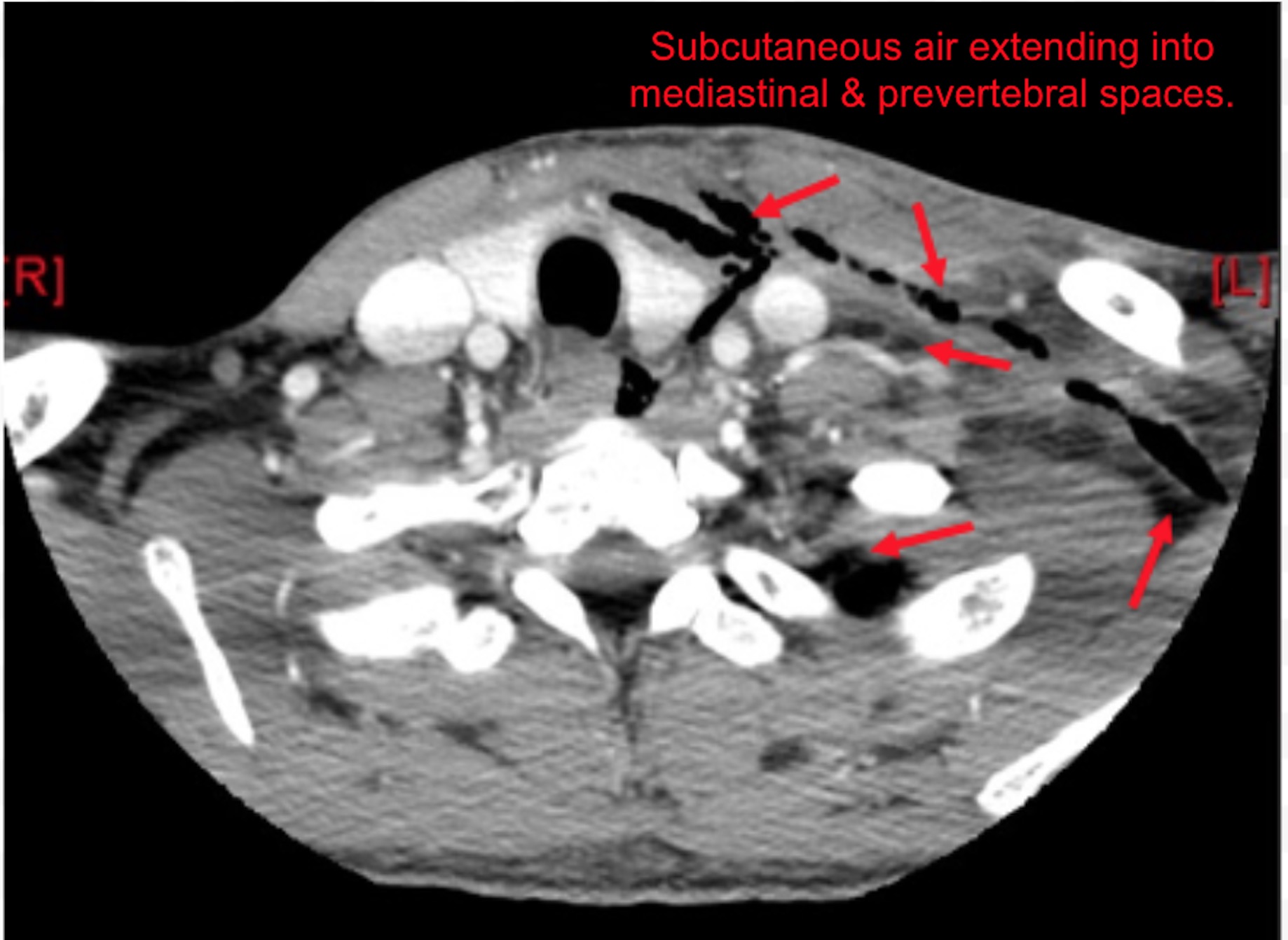

Supplement: Supplementary file 1 [file jetem-5-4-v1-supp1.jpg]

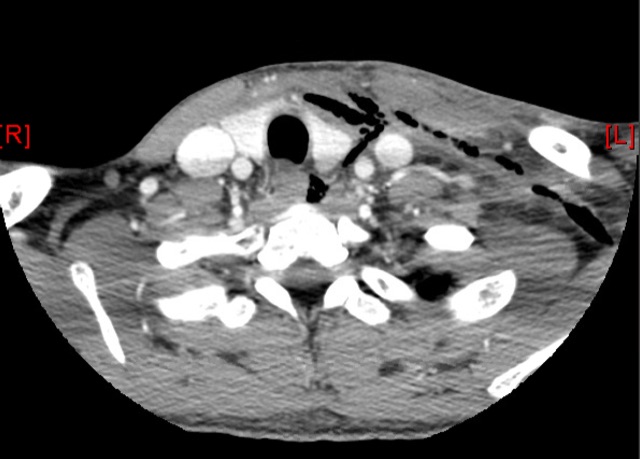

Supplement: Supplementary file 2 [file jetem-5-4-v1-supp2.jpg]

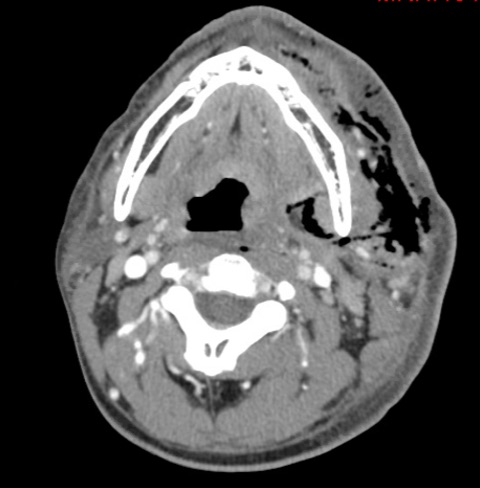

Supplement: Supplementary file 4 [file jetem-5-4-v1-supp4.jpg]

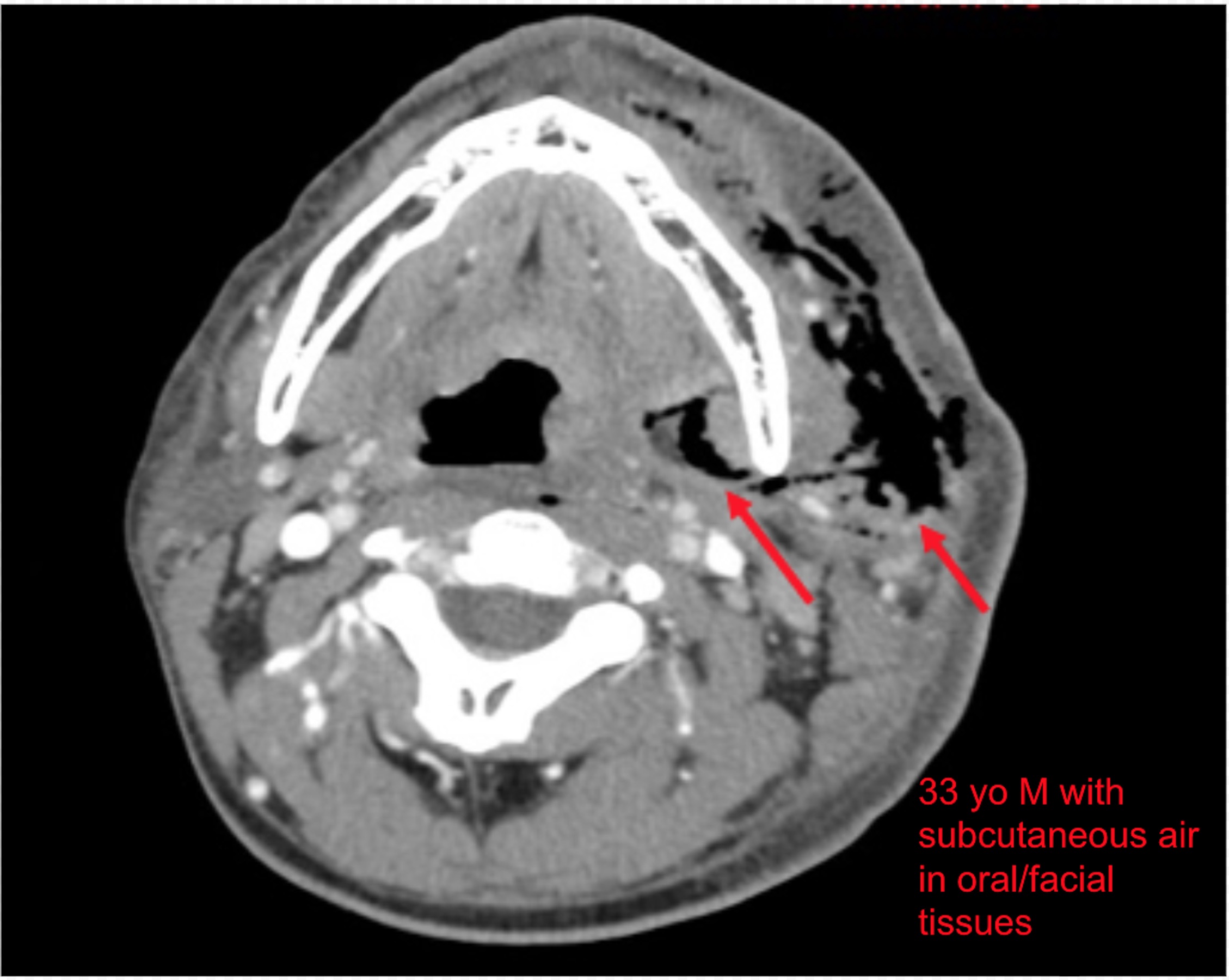

Supplement: Supplementary file 5 [file jetem-5-4-v1-supp5.jpg]
